# Verteporfin photodynamic therapy of retinal capillary hemangioblastoma in von Hippel-Lindau disease

**DOI:** 10.4103/0301-4738.58479

**Published:** 2010

**Authors:** Harsha Bhattacharjee, Hemalata Deka, Satyen Deka, Manab Jyoti Barman, Mrinal Mazumdar, Jnanankar Medhi

**Affiliations:** Vitreo-Retinal Services, Sri Sankaradeva Nethralaya, Beltola, Guwahati-781028, Assam, India

**Keywords:** Photodynamic therapy, retinal capillary hemangioblastoma, verteporfin, von Hippel-Lindau disease

## Abstract

An 18-year-old boy presented to us with bilateral retinal hemangioblastoma and von Hippel-Lindau disease with history of cerebral capillary hemangioblastoma and embryonic cell carcinoma of left testes. The vision in the right eye was already lost with development of secondary closed angle glaucoma, optic atrophy with subsequent development of bullous keratopathy. The multiple retinal angiomatous lesions in the seeing left eye were treated with various modalities like triple freeze thaw cryopexy, focal lasers and transpupillary thermo therapy in multiple sittings over a period of almost 20 years since detection. One particular angiomatous lesion in the left eye was showing resistance to all the above mentioned modalities and was finally successfully treated with verteporfin and photodynamic therapy to achieve complete regression without any post-treatment complication and with a sustained 20/20 vision till a follow-up of 15 months.

Retinal capillary hemangioblastoma is a clinically and histopathologically distinct tumor of the retina and may occur as a part of a systemic disease, including von Hippel-Lindau (VHL) disease or as an acquired retinal vasoproliferative disorder.[1] Ophthalmoscopically, retinal hemangioblastoma has a characteristic vascular appearance often with dilated feeder vessels and may present with various clinical manifestations.

Various treatment modalities, with their inherent problems, limitations and side-effects have been described in literature.[2–4] Photodynamic therapy (PDT) with verteporfin is an approved method of treatment of choroidal neovascular membrane. PDT has also been tried in cases of retinal angiomatosis with varying success.[5–9] This report describes the treatment of a large endophytic retinal angioma in a case of VHL disease using PDT with verteporfin.

## Case Report

**Figure 1:**
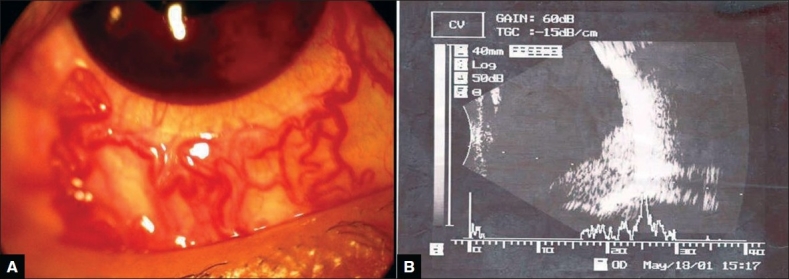
(A) Racemose dilatation of conjunctival blood vessels and note cosmetic contact lens in position. (B) USG picture showing mass lesion in inferior and infero-temporal quadrant

An 18-year-old boy with VHL disease presented to us with bilateral angiomatous retinal lesions. Examination revealed secondary closed angle glaucoma, optic atrophy and retinal angioma with subsequent development of bullous keratopathy and racemose dilatation of the conjunctival blood vessels [Fig. 1] in the right eye. Fundus was not visible because of media haziness. Ultrasound picture showed mass lesion arising from the posterior pole and extending in different directions with variability, mostly in the inferior and infero-temporal quadrant covering disc and macula [Fig. 1]. Left eye had a solitary mass of retinal angioma situated at the infero-temporal 5 o'clock position anterior to the equator with macular exudation. Triple freeze thaw cryopexy was done in the left eye with subsequent mild regression of the tumor as well as the macular edema.

He had developed right cerebral capillary hemangioblastoma with neurological symptoms in 1981, which was excised surgically. In 1993, the patient developed embryonic cell carcinoma in the left testes with metastases to the posterior cranial fossa and both lungs. Left orchidectomy was done and the patient received full course of chemotherapy with bleomycin, etoposide, and cisplatin in standard dosage and subsequently he recovered. Between 1994 and 1995, during chemotherapy, two new retinal angiomas appeared in the left eye and were kept under close observation. In 2000, he developed posterior vitreous detachment (PVD), foveo-macular traction, macular pseudo-hole in the left eye which spontaneously subsided

The inferior temporal capillary hemangioblastoma in the left eye was first detected and treated by triple freeze thaw cryopexy (two sessions) in 1987 elsewhere. At the time of detection it was a 2.5-mm size hemangioblastoma. He was subsequently treated again at our institute with triple freeze thaw cryopexy (Frigitronics, with retinal cryo probe) in 2001 (single session), 2004 (two sessions) and 2006 (single session). In between focal lasers (Zeiss Frequency Doubled NdYag laser- 532 nm) were done in 2002 and 2004. Transpupillary thermo therapy (TTT) was tried once in 2004 and again in 2006 (Nidek Diode Laser, 2000 spot size 600 mw 120 second exposure). This combination was tried during the period of 2001-06 for that particular infero-temporal capillary hemangioblastoma as it was showing an erratic growth pattern of progression and relative regression in size.

**Figure 2:**
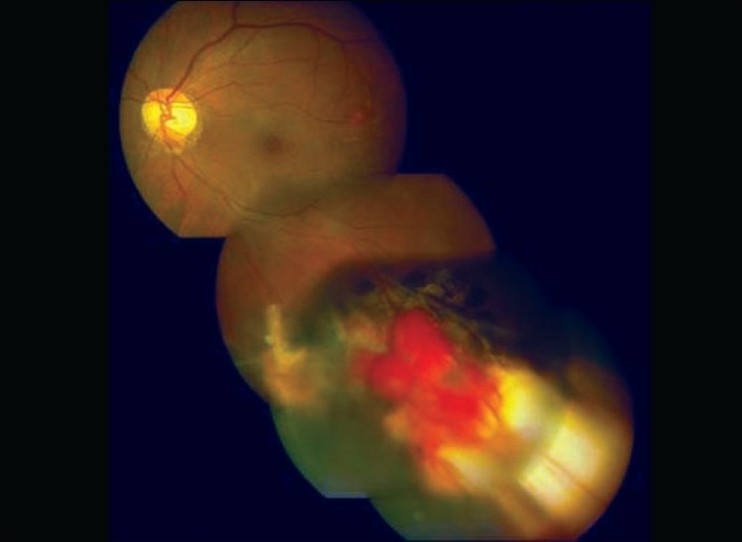
Growing endophytic retinal hemangioblastoma after repeated cryo and laser photocoagualation

On a subsequent examination in 2006, it was found that the lesion enlarged endophytically in a multi-lobulated fashion to attain a size of about 8.5 × 2.97 × 3.0 cu mm on ultrasound [Fig. 2] and it was decided to treat it by PDT with verteporfin.

The other hemangioblastoma which had developed in the left eye between 2002 and 2004 were treated at the very initial stage with focal laser (Zeiss Frequency Doubled NdYag laser- 532 nm) and these regressed satisfactorily.

**Figure 3:**
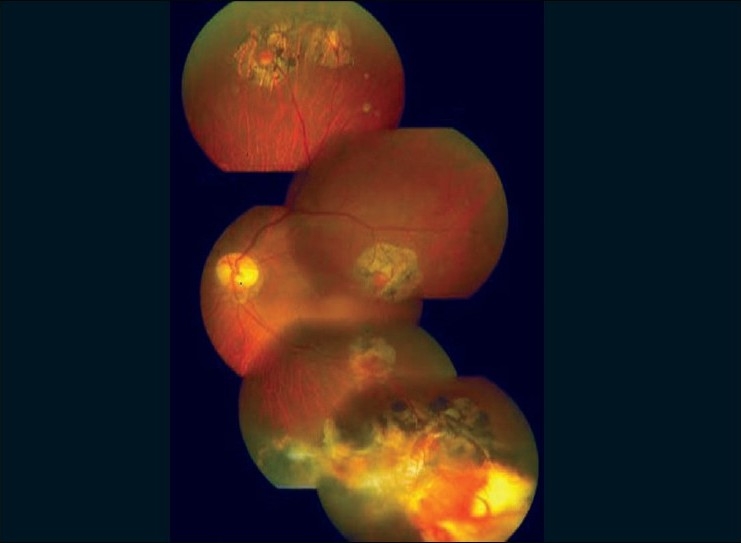
One week after PDT showing regression of the retinal hemangioblastoma. Four other small regressed hemangioblastoma are also seen

**Figure 4:**
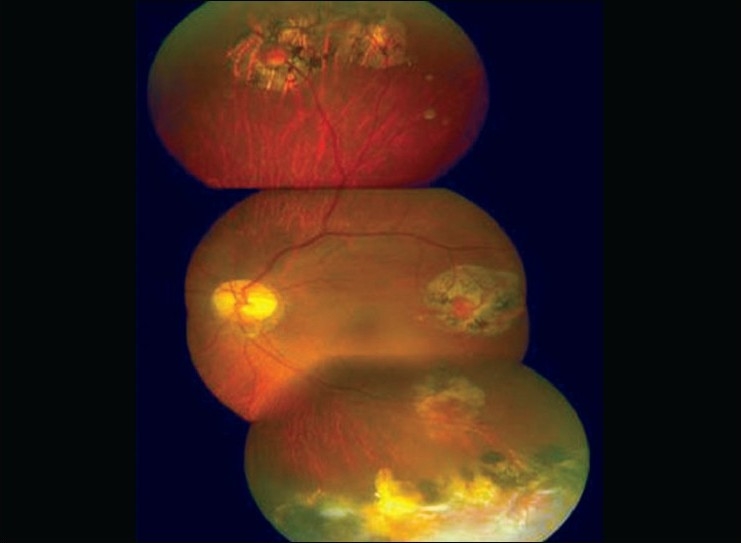
Completely regressed and scarred hemangioblastoma after six months

Intravenous verteporfin (6 mg/m^2^ body surface area) was administered over 10 min and 15 min after the start of infusion, laser at 680 nm was delivered using the Mainstar wide field lens at an intensity of 600mW/cm^2^ over 83 sec (power 50 J/cm^2^). Five successive confluent burns of 7300μ spot size (GLD) each were applied. Tumor regression was noted by the end of the first week, which continued, and by the end of 12 weeks the lesion became flat and fibrotic with surrounding dilated capillaries [Fig. 3]. Six months later the lesion became completely fibrosed [Fig. 4]. The other four angiomas continued to remain in the regressed state till the last follow-up after 15 months. The patient had a flat retina with 20/20 vision without any recurrence. Periodic routine screening excluded any pancreatic or adrenal angiomas.

## Discussion

PDT with verteporfin has been known to be a safe and effective treatment of sub-retinal neovascularization. This therapy has been tried in cases of angiomatosis retinae in VHL disease.[5–8] It has been hypothesized that as verteporfin targets vascular endothelial cells,[9,10] there may be selective affinity for verteporfin in the leading pathological capillaries of hemangioma.

Acquired vasoproliferative lesions of the retina have been associated with a wide variety of conditions leading to ischemia, inflammation or both. This in turn presumably releases vasoproliferative cytokines. Along with the growth of the blood vessels in the acquired vasoproliferative lesion, subretinal exudation and subsequent retinal detachment have been noted. It stimulates outer retinal ischemia and further release of vascular endothelial growth factors, creating a self-reinforcing cycle. Histopathologically, capillary hemangioblastoma is composed of capillary blood-filled spaces lined by endothelial cells and pericytes. In between capillaries, characteristic foamy, pale polygonal stromal cells, which stain positively for glial fibrillary, acidic protein and neuron-specific enolase, are seen. Deleted VHL gene may be restricted to stromal cells, suggesting that the stromal cells are the neoplastic component in the retinal hemangioma and may induce the accompanying neovascularization. This explained another probable mechanism of action of PDT in VHL disease.

VHL is an inherited cancer syndrome characterized by a predisposition to development of multiple retinal angiomas, cerebellar hemangiomas, bilateral renal cysts and carcinoma, bilateral pheochromocytoma, pancreatic cyst and epididymal cyst. However, the present case had associated embryonic carcinoma of left testes with distant metastases, which so far, has not been reported in literature. The new retinal angiomas appeared to develop even when the patient was on cancer chemotherapy, suggesting bleomycin (glycopeptide antibiotics), etoposide (semi-synthetic derivatives of podophyllotoxin) and cisplatin (platinum coordination complex) had no inhibitory effect on the genesis of retinal hemangioblastoma.

Pubmed search revealed seven cases of retinal angiomas having encouraging results after being treated by PDT. Coleman *et al.*[8] have reported two cases where PDT and non-thermal laser were used and additional scatter laser photocoagualation on the non-perfused retina was done in one of them. In both the cases, the angiomas regressed and closed. Similarly, Bakri *et al.*[5] reported one case where retinal hemangioma was successfully closed after verteporfin PDT along with argon laser and TTT. However, the patient developed a combined rhegmatogenous and exudative retinal detachment with proliferative vitreo-retinopathy that needed subsequent vitrectomy, scleral buckling and silicone oil tamponade.

In the present case a large retinal angioma, measuring 8.5×2.97×3.0 cu mm, resistant to cryopexy, laser photocoagulation (532 nm) and TTT was successfully treated with verteporfin and PDT with five confluent burns. The angioma regressed completely, became fibrosed without any post-treatment complication with a sustained 20/20 vision. Owing to the nature of the disease, new retinal angiomas may appear through a person's lifetime. However, PDT with verteporfin can be recommended as an effective treatment modality for individual retinal angiomas in VHL disease.
